# Four new species of the genus *Philanthaxia* Deyrolle, 1864 from Southeast Asia and comments on *P. iris* Obenberger, 1938 (Coleoptera, Buprestidae, Thomassetiini)

**DOI:** 10.3897/zookeys.116.1403

**Published:** 2011-07-07

**Authors:** Svatopluk Bílý, Oto Nakládal

**Affiliations:** Czech University of Life Sciences Prague, Faculty of Forestry and Wood Sciences, Department of Forest Protection and Game Management, Kamýcká 1176, Praha 6 – Suchdol, CZ-165 21, Czech Republic

**Keywords:** Taxonomy, Coleoptera, Buprestidae, Thomassetiini, *Philanthaxia*, new species, sexual dimorphism, Oriental region

## Abstract

Descriptions of four new species of the genus *Philanthaxia* Deyrolle, 1864: *Philanthaxia pseudoaenea* **sp. n.** (Thailand), *Philanthaxia jakli* **sp. n.** (Indonesia, Sumatra), *Philanthaxia chalcogenioides* **sp. n.** (Indonesia, Sabah) and *Philanthaxia lombokana* **sp. n.** (Indonesia, Lombok) are given. The new species and male genitalia are illustrated and compared with the most similar congeners. Sexual dimorphism of *Philanthaxia iris* Obenberger, 1938 is described and discussed.

## Introduction

The genus *Philanthaxia* was described by Deyrolle (1864) with *Philanthaxia curta* Deyrolle, 1864 as a type species (by monotypy). The genus was revised by Bílý (1993) and a further five species later described by Bílý (1997). Novak (1994) described the male of *Philanthaxia rufimarginata* (Saunders, 1866). The genus was placed to the tribe Thomassetiini Bellamy, 1987 by Bílý (2000). Additional species were subsequently described by (Bílý (2001, 2006). Currently the genus is comprised of 61 species with primarily Oriental distribution except for two species extending into the Australasian region (Bellamy 2008). This study describes another four species and the genus now contains 65 species.

An Olympus SZX 12 microscope with a fixed camera was used to capture the colour images and a MBS-10 stereoscopic microscope for drawings. Data from locality labels are cited “verbatim”. The following codens of institutional and private collections are used in the text:

NMPC	National Museum, Prague, Czech Republic

NSMT	National Science Museum Tokyo, Japan

SJCP	Stanislav Jákl collection, Prague, Czech Republic

SOCT	Sadahiro Ohmomo collection, Tsukuba, Japan

## Taxonomy

### Philanthaxia pseudoaenea sp. n.

urn:lsid:zoobank.org:act:A524A437-87D3-4354-B46A-4177C0BAA7BE

http://species-id.net/wiki/Philanthaxia_pseudoaenea

Figs 1 6 

#### Diagnosis.

**Figures 1–5.**
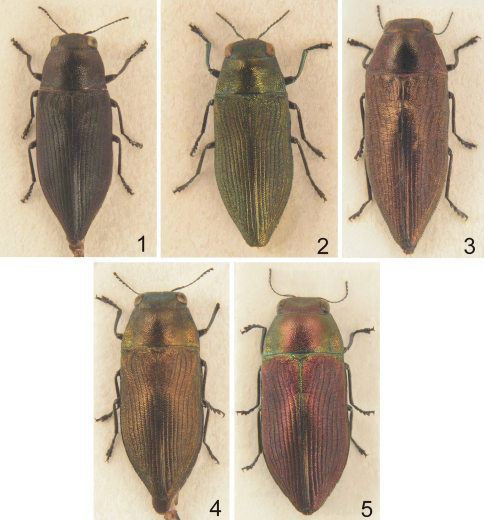
*Philanthaxia*, habitus, dorsal view **1** *Philanthaxia pseudoaenea* sp. n., holotype, 8.1 mm **2** *Philanthaxia jakli* sp. n., holotype, 7.5 mm **3** *Philanthaxia chalcogenoides* sp. n., holotype, 10.0 mm **4** *Philanthaxia lombokana* sp. n., holotype – male, 8.0 mm **5** *Philanthaxia lombokana* sp. n., paratype – female, 10.0 mm.

 Medium-sized (6.2–9.8 mm), subelliptical, moderately convex; dorsal surface black-bronze (male Fig. 1) or black-bronze with violet tinge more distinct along lateral margins, at basal portion of elytra and along anterior half of elytral suture (female); frons, antennae and legs black-violet; ventral surface black-violet, abdominal ventrites bronze; dorsal surface entirely asetose, ventral surface with very fine, sparse, recumbent, white pubescence which is somewhat denser at posterior angles of abdominal ventrites; lateral margin of metacoxae with small patch of white tomentum.

#### Description of the holotype.

 Head large, distinctly wider than anterior pronotal margin; frontoclypeus widely, shallowly emarginate separated from frons by rather deep, transverse impression; frons flat with shallow, triangular depression and wide, rounded tubercle above frontoclypeal line; vertex very slightly convex, 3.5 times as wide as width of eye; eyes relatively small, nearly elliptical, slightly projecting beyond outline of head; antennae long, slender, reaching posterior third of lateral pronotal margins when laid alongside; scape 4.5 times as long as wide, slightly claviform, nearly straight; pedicel ovoid, 1.5 times as long as wide; third antennomere very small, nearly cylindrical, 1.5 times as long as wide; antennomeres 4–10 obtusely triangular, 1.2–1.5 times as long as wide; terminal antennomere rhomboid, slightly longer than wide; sculpture of frons consisting of small, very dense oval cells without central grains, that of vertex much finer.

Pronotum distinctly bell-shaped, 1.7 times as wide as long, regularly convex; lateroposterior depressions indistinct; anterior margin shallowly biarcuate, posterior margin nearly straight; lateral margins S-shaped, posterior angles sharp; maximum pronotal width at base; lateral pronotal keel S-shaped, not visible from above, its middle portion straight; sculpture consisting of small, very dense, polygonal cells which are transversely widened at prescutellar portion and fused in lateroposterior depressions. Scutellum small, flat, obtusely pentagonal, twice as wide as long.

Elytra regularly convex, subparallel at anterior three fourths, 1.9 times as long as wide; apical fourth sinuously tapering posteriorly, slightly caudiform, very finely serrate; humeral swellings small; basal, transverse depression deep at humeral swellings, shallow towards scutellum; elytral epipleura well-developed, tapering posteriorly, reaching posterior fifth of elytra; each elytron with eight fine striae; interstices with fine, dense, transverse rugae.

Ventral surface finely, densely punctato-ocellate, abdominal ventrites rather lustrous, very finely ocellate; prosternal process weakly convex, subparallel, obtusely pointed apically; anal ventrite narrowly rounded apically, very finely serrate laterally with weak medial, longitudinal elevation at apical half. Legs long, slender, meso- and metatibiae not modified. Tarsal claws robust, strongly hook-shaped with large, basal tooth.

**Figures 6–11.**
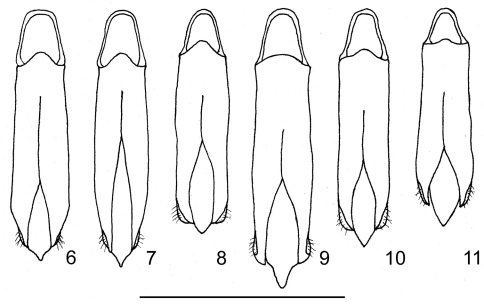
Male genitalia **6** *Philanthaxia pseudoaenea* sp. n., holotype **7** *Philanthaxia aenea* (Saunders, 1866), (Thailand, Petchaburi) **8** *Philanthaxia jakli* sp. n., holotype **9** *Philanthaxia chalcogenoides* sp. n., holotype **10** *Philanthaxia lombokana* sp. n., holotype **11** *Philanthaxia iris* Obenberger, 1938 (Java, Watu Ulo). Scale bar: 2 mm.

Aedeagus (Fig. 6) flat, subparallel, parameres somewhat enlarged and swollen on apical fourth, strongly narrowed apically.

Sexual dimorphism. Female differs from male only by the somewhat more robust body and more distinct violet tinge along the anterior and lateral margins of elytra.

Variability. Except for the size and sexual dimorphism only a slight variability was observed in colouration: some specimens are nearly black only with very weak bronze tinge.

Measurements. Length: 6.2–9.8 mm (holotype 8.1 mm); width: 2.3–3.4 mm (holotype 2.8 mm).

#### Type specimens.

 Holotype (male, NSMT): “NE Thailand, Pak Chong, Korate, Kasetsart Farm, 13.v.2008, S. Ohmomo leg.”; paratypes (30 males, 17 females, NMPC, NSMT, SOCT): the same data (24 males, 12 females); “NE Thailand, Saraburi, Phra Buddaha Chai, 14.v.2008, S. Ohmomo leg.” (6 males, 4 females).

#### Type locality.

Northeastern Thailand, Pak Chong, Korate, Kasetsart Farm.

#### Etymology.

 The specific epithet “*pseudoaenea*” expresses the similarity with the sympatric species, *Philanthaxia aenea* (Saunders, 1867).

#### Distribution.

 Central and northeastern Thailand, Pak Chong province.

#### Differential diagnosis.

 *Philanthaxia pseudoaenea* sp. n. belongs to the group of species with flat or impressed frons; wide scutellum and strongly toothed tarsal claws. From the very similar *Philanthaxia aenea* it differs by the characters given in Table 1.

**Table 1.** Comparison between *Philanthaxia pseudoaenea* sp. n. and *Philanthaxia aenea*.

*Philanthaxia pseudoaenea* sp. n.	*Philanthaxia aenea* (Saunders, 1866)
Darker, less aeneous often with violet tinge	More aeneous without violet tinge
Pronotum distinctly bell-shaped, finely emarginate before posterior angles	Pronotum not bell-shaped, nearly straight before posterior angles
Elytra subparallel at anterior three fourths, nearly sinuously tapering at posterior fourth, slightly caudiform apically	Elytra regularly, tapering posteriorly, not caudiform apically
Aedeagus (Fig. 6) shorter, parameres somewhat enlarged and swollen at apical fourth	Aedeagus (Fig. 7) longer, parameres not enlarged at apical fourth

### Philanthaxia jakli sp. n.

urn:lsid:zoobank.org:act:CF3AD301-A880-40BC-9879-DBE355FC7F74

http://species-id.net/wiki/Philanthaxia_jakli

Figs 2 8 

#### Diagnosis.

 Medium-sized (7.5–8.0 mm), regularly convex, subparallel; dorsal surface golden green, matt; elytral margins and posterior pronotal angles and frons sometimes with red lustre; legs and scutellum green; ventral surface dark golden-green; dorsal surface asetose, ventral surface with short, recumbent, rather dense, white pubescence; lateral margins of abdominal ventrites with small patches of white tomentum.

#### Description of the holotype.

 Head large, distinctly wider than anterior pronotal margin; frontoclypeus widely, shallowly emarginate, separated from frons by shallow, transverse depression; frons flat, vertex very wide, 5 times as wide as width of eye; eyes small, nearly elliptical, strongly projecting beyond outline of head; antennae long, slender, reaching posterior third of lateral pronotal margins when laid alongside; scape slender, slightly claviform, 5 times as long as wide; pedicel ovoid, slightly longer than wide; third antennomere weakly enlarged apically, 1.5 times as long as wide; antennomeres 4–10 obtusely triangular, about 1.3 times as long as wide; terminal antennomere rhomboid, nearly twice as long as wide; sculpture consisting of very fine, dense, polygonal to oval cells with lustrous bottom.

Pronotum regularly convex with wide, shallow lateroposterior depressions, twice as wide as long; anterior margin shallowly biarcuate, posterior margin nearly straight; lateral margins nearly straight, very weakly emarginate anteriad sharp lateroposterior angles; maximum pronotal width at base; sculpture consisting of deep, dense punctures at posterior angles, fine, dense polygonal cells at anterior third and fine, dense, transverse rugae on disc and prescutellar portion. Scutellum lustrous, widely pentagonal, 2.2 times as wide as long.

Elytra regularly convex, subparallel at anterior two thirds, narrowly tapering at posterior third, 2.1 times as long as wide; posterior third only indistinctly caudiform, very finely serrate; humeral swellings small; basal transverse depression developed only on humeri; elytral epipleura narrow, reaching posterior third of elytra; each elytron with eight fine striae, interstices with fine, dense, transverse rugae.

Ventral surface lustrous, finely, densely ocellate; prosternal process flat, obtusely pointed apically; anal ventrite nearly flat, obtusely truncate apically, without distinct lateral serration. Legs long, slender, meso- and metatibiae straight. Tarsal claws thin, strongly hook-shaped, slightly enlarged at base.

Aedeagus (Fig. 8) wide, flattened, parallel-sided; apical portion of parameres angulately rounded, median lobe widely pointed.

Female unknown.

Variability. The paratype possesses a more distinct golden tinge along the elytral margins, at the posterior pronotal angles and on the frons than the holotype.

Measurements. Length 7.5 mm (holotype) and 8.0 mm (paratype); width: 2.6 mm (holotype), 2.9 mm (paratype).

#### Type specimens.

 Holotype (male, NMPC): “Indonesia, West Sumatra, Harau valley env., 500–800m, cca 20km N of Payakumbuh, iv.-v.2006, St. Jákl leg.”; 1 paratype (male, NMPC): the same data except for “600m, v.2007”.

#### Type locality.

 Indonesia, West Sumatra, Harau valley, 500–800 m, cca 20 km N of Payakumbuh.

#### Etymology.

 This species is dedicated to the collector, S. Jákl (Prague, Czech Republic).

#### Distribution.

 Western Sumatra, prov. Aceh.

#### Differential diagnosis.

 *Philanthaxia jakli* sp. n. belongs to the group of species with flat or impressed frons, wide scutellum, simple tarsal claws and golden green dorsal colouration. Accordingly to the key (Bílý 2001), *Philanthaxia jakli* sp. n. should stand in one couplet with *Philanthaxia similis* Bílý, 2001 from Laos but it differs from it by having a much more slender body, darker colouration, pentagonal scutellum (cordiform in *Philanthaxia similis*), flat frons (roundly impressed in *Philanthaxia similis*) and by the shape of male genitalia (aedeagus regularly tapering posteriorly and parameres regularly rounded apically in *Philanthaxia similis*). It is similar to *Philanthaxia acuminata* Bílý, 1993 from Borneo (Sabah) but it differs from this species by the colouration (reddish-coppery with golden-red frons in *Philanthaxia acuminata*), shape of scutellum (wider and deeply impressed in *Philanthaxia acuminata*) and by the shape of male genitalia (aedeagus longer, not parallel-sided with slightly enlarged parameres at apical third in *Philanthaxia acuminata*). From the sympatric species, *Philanthaxia sumatrensis* Bílý, 1993 it differs by the absence of golden-purple elytral stripes, slender body and by the different male genitalia (spindle shaped aedeagus in *Philanthaxia sumatrensis*).

### Philanthaxia chalcogenoides sp. n.

urn:lsid:zoobank.org:act:D61A7895-186A-408B-BE14-2CA8396CA981

http://species-id.net/wiki/Philanthaxia_chalcogenoides

Figs 3 9 

#### Diagnosis.

 Large (10.0 mm) lustrous, convex; dorsal surface bright bronze, medial portion of pronotum somewhat darkened, elytra lustrous along suture with distinct mirror-effect (Fig. 3); scutellum with purple lustre; ventral surface bronze, prosternal process and middle portion of metasternum lustrous; dorsal surface entirely asetose, ventral surface with extremely fine, sparse, white pubescence.

#### Description of the holotype.

 Head as wide as anterior pronotal margin; frontoclypeus widely, shallowly emarginate, separated from frons by deep, transverse impression; frons flat with shallow, rounded impression at middle; vertex 4 times as wide as width of eye; eyes small, ellyptical, slightly projecting beyond outline of head; antennae long and slender, reaching posterior fourth of lateral pronotal margins when laid alongside; scape nearly straight, slightly claviform, 5 times as long as wide; pedicel ovoid, 1.6 times as long as wide; third antennomere very small, slender, nearly twice as long as wide; antennomeres 4–10 obtusely triangular to trapezoidal, 1.3–1.6 times as long as wide; terminal antennomere rhomboid, slightly longer than wide; sculpture consisting of small, very dense, oval cells with lustrous bottom; cells in frontal impression slightly prolonged vertically.

Pronotum rather convex, flattened at prescutellar portion, twice as wide as long; both anterior and postrior margins very weakly biarcuate; lateral margins very slightly rounded, nearly straight, posterior angles sharp; lateroposterior depressions indistinct, maximum pronotal width at base; sculpture consisting of small, fine, simple punctures on disc and small, rather deep, dense, polygonal cells on lateral sides. Scutellum subtriangular, very lustrous, twice as wide as long.

Elytra convex, twice as long as wide, subparallel at anterior two thirds; posterior third regularly acuminate posteriorly with finely, densely serrate margins; humeral swellings well-developed; basal, transverse depression developed only on lateral half of elytra; elytral epipleura very narrow, reaching posterior third of elytra; each elytron with eight, very fine striae; interstices flat with fine, dense, transverse rugae.

Ventral surface very densely, finely ocellate, prosternal process flat, weakly enlarged posteriad procoxae, obtusely pointed apically; anal ventrite weakly convex, without distinct lateral serration, shortly truncate to emarginate apically. Legs long, slender, meso- and metatibiae straight. Tarsal claws small, strongly hook-shaped, only weakly enlarged at base.

Aedeagus (Fig. 9) short, robust, flattened, widely spindle-shaped; median lobe sharply pointed apically.

Female unknown.

Measurements. Length: 10.0 mm; width: 3.6 mm.

#### Type specimen.

 Holotype (male, NMPC): “Malaysia, Sabah, Crocker Range, vic. of Mt. Trus-Madi, iii.-iv.2002, local collector”.

#### Type locality.

 Malaysia, Sabah, Crocker Range, vic. of Mt. Trus-Madi.

#### Distribution.

 Borneo: Sabah province.

#### Etymology.

 The specific epithet refers to the superficial similarity to species of the genus *Chalcogenia* Saunders, 1871.

#### Differential diagnosis.

 *Philanthaxia chalcogenoides* sp. n. resembles some species of the genus *Chalcogenia* (Anthaxiini) by the body-shape, colouration and by distinct elytral mirror effect. It differs from its congeners by the strange pronotal sculpture (deeply rugate or exceptionally deeply ocellate in other species) and by the conspicuous mirror effect along the elytral suture. With the shape and colouration, it resembles *Philanthaxia akiyamai* Bílý, 1993 described from the Peninsular Malaysia (but recently collected in Sabah). The latter species differs from *Philanthaxia chalcogenoides* sp. n. by the rough, rugose pronotal sculpture; matt dorsal surface; wider scutellum and by the shape of male genitalia (Fig. 9).

### Philanthaxia lombokana sp. n.

urn:lsid:zoobank.org:act:000A3B8D-6886-4890-A6CA-B94F8A5D23B6

http://species-id.net/wiki/Philanthaxia_lombokana

Figs 4, 5 10 

#### Diagnosis.

 Rather large (6.7–10.1 mm), robust, strongly convex with silky lustre; dorsal surface of male (Fig. 4) bronze with feeble purple lustre; elytra with slightly developed mirror effect at posterior half; frons golden-green, vertex bronze; dorsal surface of female (Fig. 5) purple-bronze, lateral portion of elytra dark blue-violet; scutellum golden green, narrow postscutellar, sutural stripe golden; antennae and legs black with weak bronze lustre in both sexes; ventral surface black with brass tinge; dorsal surface asetose; ventral surface with sparse, fine, recumbent, white pubescence; prosternal process with rather dense, erect, grey pubescence; distal portion of metacoxae and lateral portions of abdominal ventrites with patches of white tomentum.

#### Description of the holotype.

 Head large, distinctly wider than anterior pronotal margin; frontoclypeus widely, shallowly emarginate; frons flat with shallow, rounded, medial impression; vertex flat, 5 times as wide as width of eye; eyes relatively small, elliptical, rather strongly projecting beyond outline of head; antennae slender, reaching posterior third of lateral pronotal margins when laid alongside; scape very slender, slightly curved, 6 times as long as wide; pedicel small, ovoid, 1.8 times as long as wide; third antennomere small, slightly claviform, 1.6 times as long as wide; antennomeres 4–10 obtusely triangular, 1.2–1.8 times as long as wide; terminal antennomere rhomboid, 1.8 times as long as wide; sculpture consisting of small, very dense, oval or rounded cells without central grains which are larger and more prolonged in frontal impression, smaller and rounded on remainder of head.

Pronotum strongly convex, nearly bell-shaped, 1.9 times as wide as long; lateroposterior depressions nearly indistinct; anterior margin shallowly biarcuate, posterior margin nearly straight; lateral margins straight, tapering anteriorly at anterior half, regularly arched at posterior half; posterior angles nearly rectangular; maximum pronotal width at base; sculpture consisting of small, dense, rounded cells without central grains on anterior half; prescutellar portion with rather rough, long, transverse rugae, posterior angles with short, transverse rugae. Scutellum widely pentagonal, slightly impressed medially, 2.5 times as wide as long.

Elytra regularly, strongly convex, 1.8 times as long as wide with maximum width at posterior third, slightly caudiform; humeral swellings well-developed; basal, transverse depression short, wide, not reaching scutellum; elytral epipleura narrow, reaching posterior third of elytral length; posterior third of elytral margins weakly serrate; each elytron with eight fine striae; interstices with fine, short, transverse rugae at posterior half; basal half of elytra with rough, deep, transverse rugae.

Ventral surface rather lustrous, finely ocellate, prosternum roughly ocellate; prosternal process very weakly convex, obtusely pointed apically, not enlarged posteriad procoxae; anal ventrite apically obtusely rounded, without lateral serration. Legs relatively long, slender; meso- and metatibiae finely bent outwards at posterior half. Claws slender, hook-shaped, slightly enlarged at base.

Aedeagus (Fig. 10) short, flat, nearly spatulate, parameres subparallel at basal half, slightly enlarged and regularly rounded towards apex at posterior half; median lobe simply pointed apically.

Sexual dimorphism. Except for the colouration (see above) female differs from male only by more robust and larger body.

Variability. Pronotum 1.9–2.1 times as wide as long; elytra 1.7–1.9 times as long as wide. No variability in colouration was observed in males; several female paratypes possesses darker lateral portion of elytra (nearly black with slight metallic tinge) and darkened medial portion of pronotum (black with intensive blue-green tinge).

Measurements. Length 6.7–10.1 mm (holotype 8.0 mm); width: 2.6–4.0 mm (holotype 3.0 mm).

#### Type specimens.

 Holotype (male, NMPC): “Indonesia, Lesser Sundas, Lombok Isl., xi.2008, S slopes of Mt. Rinjani, 800 m, local collectors”; paratypes (19 males, 23 females, NMPC, SJCP): the same data.

#### Type locality.

 Indonesia, Lesser Sundas, Lombok Isl., S slopes of Mt. Rinjani, 800 m.

#### Etymology.

 This species is named after the locality of the type specimens, Lombok Island.

#### Distribution.

 Indonesia, Lombok Island

#### Differential diagnosis.

 *Philanthaxia lombokana* sp. n. belongs to the group of species with simple tarsal claws, wide depressed frons and wide scutellum. It is the third species distributed eastwards of the Wallace Line. This species is very similar to *Philanthaxia iris* Obenberger, 1938 (see below) from Java from which it differs by the different colouration, less lustrous body, more flattened frons which is only slightly medially impressed and by the different male genitalia (Figs 10 vs. 11).

### Philanthaxia iris

Obenberger, 1938

http://species-id.net/wiki/Philanthaxia_iris

Fig. 11 

#### Remarks.

This species was described by Obenberger (1938) from the single female specimen labeled “Java, Popoh, Kediri Res.”. At the time of the last revision of *Philanthaxia* (Bílý 2001), the male of *Philanthaxia iris* was unknown. Quite recently we have studied two specimens of *Philanthaxia iris* (male and female) from “Watu Ulo” (Java, local collector) so it was possible to study the sexual dimorphism.

#### Description.

The male is much smaller (6.7 mm) and nearly completely dark (black with blue-green tinge and golden green scutellum); aedeagus as in Fig. 11. The female is much larger and more robust (9.5–12.0 mm), dark golden-green elytra with longitudinal purple stripe extending from humeri to apex and purple lateral margin; the pronotum is dark reddish-purple with large, black-green spot on anterior half.

#### Distribution.

 Java.

## Supplementary Material

XML Treatment for Philanthaxia pseudoaenea

XML Treatment for Philanthaxia jakli

XML Treatment for Philanthaxia chalcogenoides

XML Treatment for Philanthaxia lombokana

XML Treatment for Philanthaxia iris
